# Effect of Membrane Pore Size on Membrane Fouling of Corundum Ceramic Membrane in MBR

**DOI:** 10.3390/ijerph20054558

**Published:** 2023-03-04

**Authors:** Rui Huang, Hui Pan, Xing Zheng, Chao Fan, Wenyan Si, Dongguan Bao, Shanshan Gao, Jiayu Tian

**Affiliations:** 1 School of Civil and Transportation Engineering, Hebei University of Technology, Tianjin 300401, China; 2 Guangdong GDH Water Co., Ltd., Shenzhen 518021, China; 3 School of Environment, Harbin Institute of Technology, Harbin 150090, China; 4 State Key Laboratory of Eco-Hydraulics in Northwest Arid Region, Xi’an University of Technology, Xi’an 710048, China; 5 Shanghai Hanyuan Engineering & Technology Co., Ltd., Shanghai 201400, China

**Keywords:** ceramic membrane, membrane fouling, membrane pore size, membrane preparation, membrane bioreactor

## Abstract

Ceramic membrane has emerged as a promising material to address the membrane fouling issue in membrane bioreactors (MBR). In order to optimize the structural property of ceramic membrane, four corundum ceramic membranes with the mean pore size of 0.50, 0.63, 0.80, and 1.02 μm were prepared, which were designated as C5, C7, C13, and C20, respectively. Long-term MBR experiments showed that the C7 membrane with medium pore size experienced the lowest trans-membrane pressure development rate. Both the decrease and increase of membrane pore size would lead to more severe membrane fouling in the MBR. It was also interesting that with the increase of membrane pore size, the relative proportion of cake layer resistance in total fouling resistance was gradually increased. The content of dissolved organic foulants (i.e., protein, polysaccharide and DOC) on the surface of C7 was quantified as the lowest among the different ceramic membranes. Microbial community analysis also revealed the C7 had a lower relative abundance of membrane fouling associated bacteria in its cake layer. The results clearly demonstrated that ceramic membrane fouling in MBR could be effectively alleviated through optimizing the membrane pore size, which was a key structural factor for preparation of ceramic membrane.

## 1. Introduction

Membrane bioreactor (MBR) integrates membrane separation technology into the activated sludge process, having exhibited unique advantages such as excellent effluent quality, reduced footprint, lowered sludge production and robust process operation for wastewater treatment and recovery [1,2,3]. Currently, the membranes employed in MBR are mainly made of organic polymers. Polymeric membranes are susceptible to fouling by the activated sludge and can be easily aged by chemical cleaning, leading to low sustainability in MBR operation [4,5,6].

In recent years, ceramic membranes have gained ever-increasing attention all over the world and have been evaluated in both drinking water and wastewater treatment [7,8,9,10]. It is believed that ceramic membranes can provide longer service life, higher permeability, and robust physical and chemical stability in comparison with polymeric membranes [11,12]. For example, Kimura and Uchida [13] reported intensive physical cleaning (e.g., scrubbing by granular materials) and chemical cleaning (enhanced backwash with 1000 mg/L NaClO) can be applied to the ceramic membrane MBR (CMBR), which was shown able to operate at an elevated flux (30.1 LMH) for a prolonged period. However, intensive membrane cleaning would significantly increase the operation cost and maintenance difficulties of the CMBR system. It is highly expected to alleviate membrane fouling by optimizing the intrinsic properties of the ceramic membrane [14].

It has been recognized that membrane pore size can exert a significant influence on the membrane fouling behavior of polymeric membranes. However, the reported trends were found to be inconsistent. For example, Jin et al. [15] compared the membrane fouling behavior of four different pore sizes in MBR, finding the membrane with the smallest pore size experienced the lowest trans-membrane pressure (TMP) development rate, while on the contrary, Sano et al. [16] noticed a larger pore size (0.57 μm) was beneficial to suppressing the membrane fouling development in MBR. Chang et al. [17] also reported the membrane with smaller pore size tended to form a thicker cake layer during MBR operation, thus resulting in more severe membrane fouling. Considering the suitable membrane pore size of MBR strongly depends on the membrane material [18,19], it is meaningful to optimize the pore size of the ceramic membrane for alleviating the membrane fouling in MBR.

Therefore, in this work, ceramic membranes with different pore sizes were prepared by using corundum powder with different grain sizes, and their membrane fouling behavior in MBR was systematically investigated and compared. The effect of membrane pore size on the accumulation of membrane foulants was also discussed. According to the results of this work, the optimum pore size of corundum ceramic membrane was obtained, which was shown able to significantly alleviate the membrane fouling in MBR.

## 2. Materials and Methods

### 2.1. Preparation of the Ceramic Membranes

**Figure 1:**
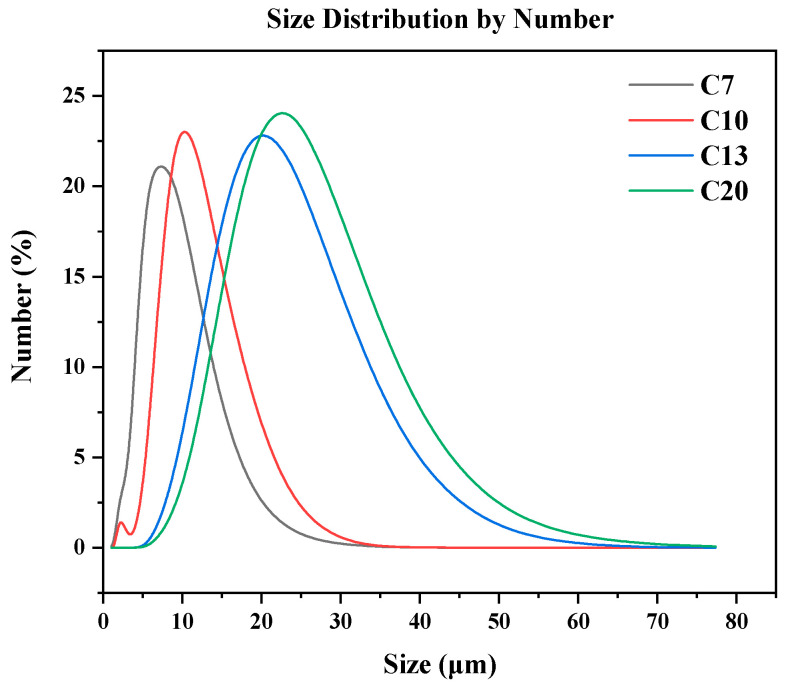
Particle size distribution of the corundum grains for preparing the ceramic membranes.

A ceramic membrane with a hollow flat-sheet configuration was prepared by extrusion molding followed by high-temperature sintering. The detailed procedure can be found in our previous study [20]. To investigate the effect of membrane pore size on membrane fouling in MBR, four corundum powders with different grain sizes (5, 7, 13, and 20 μm) were used to prepare the ceramic membranes, which were designated as C5, C7, C13, and C20, respectively. The particle size distribution of the corundum grains used for preparing the different ceramic membranes is shown in Figure 1.

### 2.2. Operation of the Laboratory-Scale MBR

**Figure 2:**
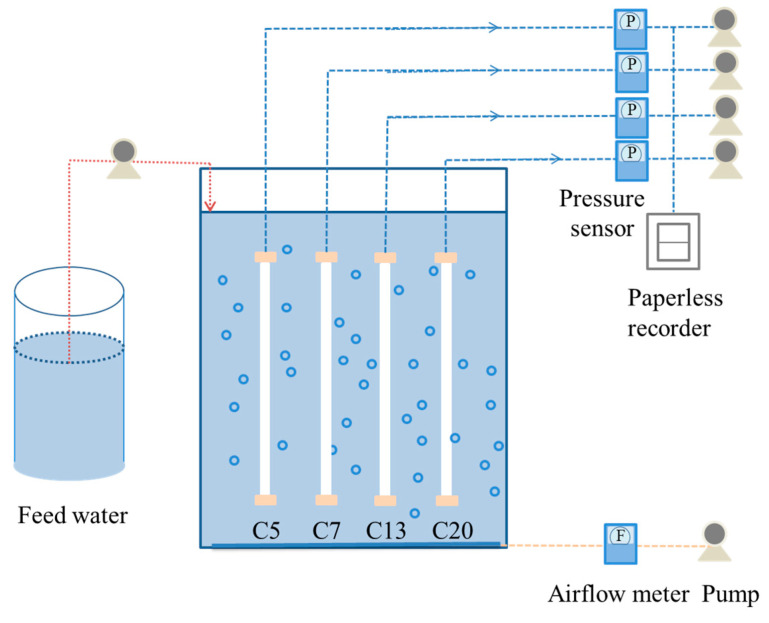
Schematic diagram of the MBR system.

The MBR system is schematically illustrated in Figure 2. The working volume of the MBR was 8.0 L with a dimensional size of 40 cm × 10 cm × 20 cm in length, width and height, respectively. In order to avoid the influence of the difference in activated sludge characteristics on membrane fouling, the four membranes with an effective area of 0.0083 m^2^ were immersed in the same bioreactor in a vertical direction, which was operated in parallel by using four individual peristaltic pumps. The operation conditions of the MBR were similar to that reported in our previous study [20].

### 2.3. Characterization of the Ceramic Membranes

The mean pore size and pore size distribution of the ceramic membranes were measured by a membrane aperture tester (3H-2000PB, Beijing Bester Instrument Technology Co., Ltd., Beijing, China). The porosity was determined according to Archimedes’ principle with the detailed procedure shown in [20]. The surface morphology of original and fouled ceramic membranes was observed by a scanning electron microscope (Hitachi SU8020 SEM, Tokyo, Japan). The phase crystal structure of the ceramic membrane was assessed by X-ray diffraction (D8 Discover, Bruker AXS, Karlsruhe, Germany).

### 2.4. Evaluation of Membrane Fouling Resistance

The membrane fouling resistance was calculated according to the resistance-in-series model, as shown in the following equations [16,21].

(1) $$R_{t}=R_{p}+R_{c}+R_{m}=\frac{\Delta P}{\mu J}$$

(2) $$R_{t}=R_{r}+R_{\mathrm{ir}}+R_{m}$$

where J is the permeation flux (L/(m^2^·h)); ∆P is the TMP (Pa); μ is the viscosity of water (Pa·s). Likewise, R_t_ is the total filtration resistance of the fouled membrane (m^−1^); R_m_ is the inherent membrane resistance (m^−1^); R_p_ is the resistance caused by membrane pore blockage (m^−1^); R_c_ is the fouling resistance of the cake layer formed on the membrane surface (m^−1^); R_r_ is the reversible fouling resistance (m^−1^); and R_ir_ is the irreversible fouling resistance (m^−1^).

### 2.5. Extraction and Analysis of Membrane Surface Foulants

The fouling layer formed on the membrane surface after MBR operation was wiped by a sponge and transferred to 50 mL of deionized water. The foulants were dispersed by ultrasonic and eddy oscillation for 5 min, respectively, and then centrifuged (10,000 r/min) for 5 min to obtain the supernatant [22]. After that, the supernatant was filtered by 0.45 μm membrane, and the concentration of protein, polysaccharide and DOC (TOC-L CPH, Shimadzu, Kyoto, Japan) were measured.

Three-dimensional fluorescence excitation-emission matrix (EEM) spectroscopy (CARY Eclipse, Agilent Technologies, Santa Clara, CA, USA) was also used to characterize the foulants extracted from the membrane surface. The spectrum was collected by changing the emission wavelength from 220 to 450 nm and the excitation wavelength from 260 to 540 nm.

### 2.6. Microbial Community Analysis

The microbial community of sludge samples extracted from the cake layer of fouled ceramic membranes and that in the mixed liquid (ML) of MBR were analyzed by Illumina MiSeq sequencing (Sangon Biotech Co., Ltd., Shanghai, China). On the V3 and V4 regions of the 16S rRNA gene, the PCR was amplified and sequenced with primers 341F (5′-TACCGGGGGGCWGCAG-3′) and 805R (5′-GACACHVGGGTATCTAATCC-3′).

## 3. Results and Discussion

### 3.1. Characteristics of the Corundum Membranes

**Figure 3:**
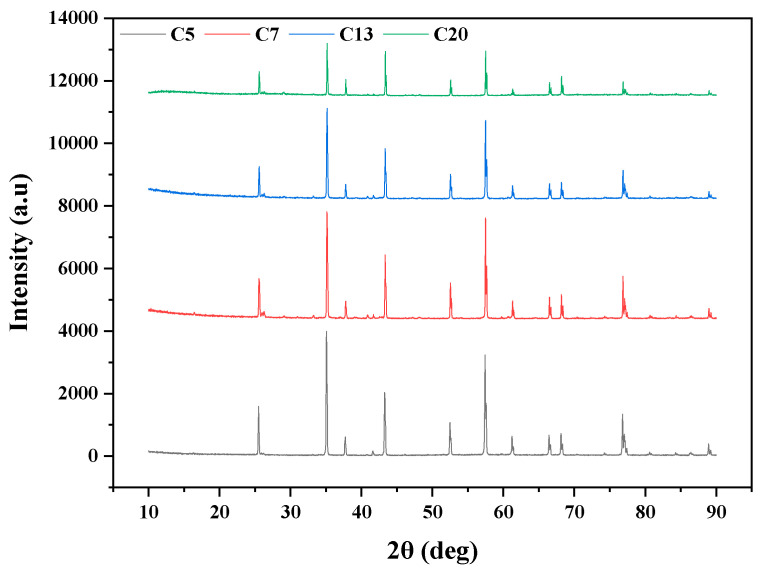
XRD of the ceramic membranes with different pore sizes.

Table 1 lists the characteristics of the ceramic membranes prepared with corundum grains of different sizes. It could be seen that the mean membrane pore size exhibited an increasing linear trend with the corundum grain size. For C5, C7, C13, and C20, the mean pore sizes were 0.50, 0.63, 0.80, and 1.02 μm, respectively. Synchronously, the porosity of the membrane also increased from 41.43% (C5) to 47.60% (C20). As a result, the pure water flux was remarkably enhanced from 2111.67 to 15,193.08 L/(m^2^·h·bar). From Figure 3, it can be seen the main crystal phase of the ceramic membrane was α-Al_2_O_3_ [23]. The position of X-ray diffraction peaks for the four membranes was essentially the same, indicating the grain size of the corundum would not influence its main crystal phase. However, it was interesting to note the microcrystal size calculated by Jade 6 was in the order of C5 < C7 < C13 < C20, which was consistent with the actual grain size of the corundum membranes.

**Figure 4:**
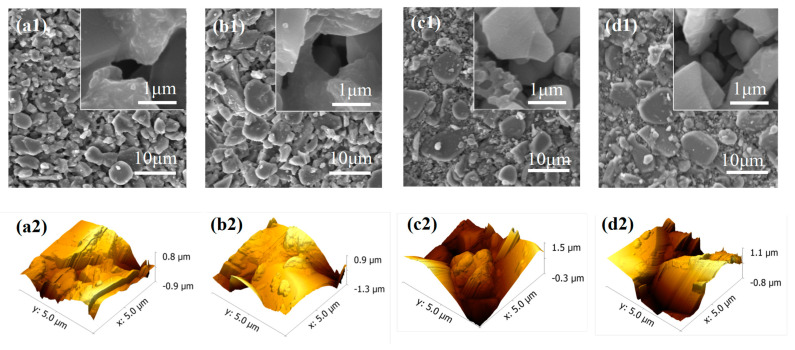

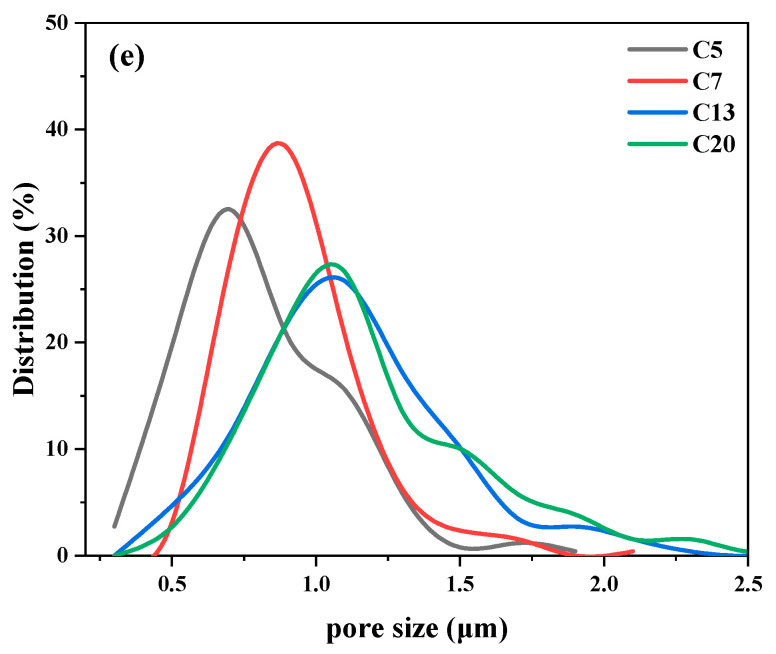
SEM and AFM images of different membranes: (**a1**–**d2**) C5, C7, C13, C20; (**e**) pore size distribution of different membranes.

Figure 4(a1–d2) shows the morphology of the corundum ceramic membranes observed by SEM and AFM. It can be seen that C5 and C7 had more regular surfaces with fewer macropore defects, which may avoid the entrance of sludge flocs into the membrane pores. With the increase in corundum grain size, more macropore defects appeared on the membrane surface, especially for the C20 membrane. This trend was further witnessed by the pore size distribution, as displayed in Figure 4e. The C5 and C7, especially the C7, possessed a narrow pore size distribution, implying the ceramic membrane prepared with a smaller corundum grain size had a more uniform pore structure [24]. By contrast, the pore size distribution of C13 and C20 was remarkably enlarged with two wide shoulders, especially on the right side, demonstrating the uneven distribution of membrane pores and the appearance of macropore defects on the membrane. The root mean square (RMS) roughness values of the four membranes were also measured, which followed the order: C20 (0.475 μm) > C13 (0.385 μm) > C7 (0.370 μm) > C5 (0.303 μm).

### 3.2. MBR Performance with Different Ceramic Membranes

**Figure 5:**
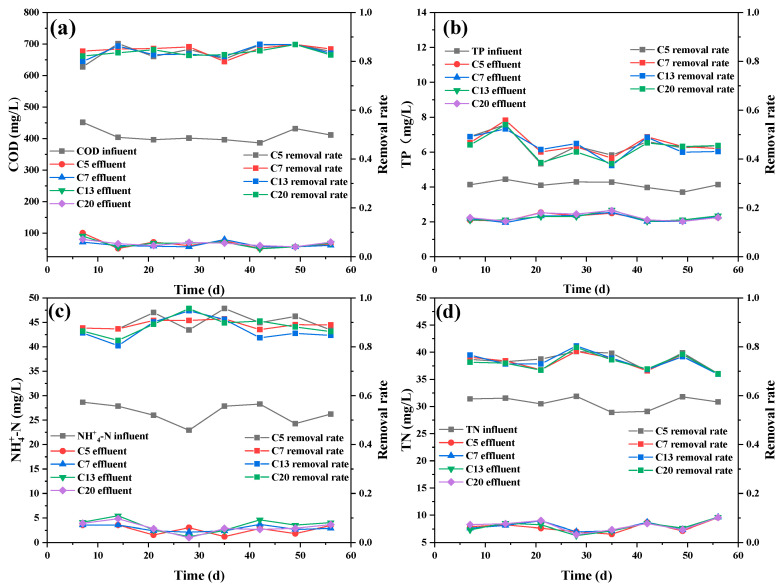
Pollutants removal in the CMBR by different membranes: (**a**) COD; (**b**) TP; (**c**) NH4^+^-N; (**d**) TN.

Figure 5a shows that the chemical oxygen demand (COD) removal in the CMBR by different membranes was approximately the same, with the removal rate higher than 83%. The high COD removal could be attributed to the high microbial activity of the activated sludge in the CMBR. In Figure 5b, it could be seen ~47% of total phosphorus (TP) removal was achieved by the CMBR, which was lower than the MBR systems reported in other studies [25,26]. The low phosphorus removal may be due to the lack of an anaerobic environment in the single-staged CMBR system, which was essential for efficient phosphorus uptake by phosphorus-accumulating organisms (PAOs) [27]. In contrast, ~88% ammonia nitrogen (NH4^+^-N) was obtained by the CMBR with different ceramic membranes (Figure 5c), which was shown to be higher than the 66.7~76.9% as reported by other researchers [28,29,30], further illustrating a high microbial activity was maintained in the CMBR. It was interesting that although a high DO of ~3 mg/L was maintained in the bioreactor, as high as 75% of total nitrogen (TN) removal efficiency was still achieved by the single-staged CMBR (Figure 5d), possibly due to the high MLSS in the bioreactor (~7500 mg/L). The high TN removal could be considered an additional advantage for the MBR system in comparison with the conventional activated sludge process [31].

### 3.3. Effect of Membrane Pore Size on Membrane Fouling

#### 3.3.1. Changes in TMP and Membrane Fouling Resistance

**Figure 6:**
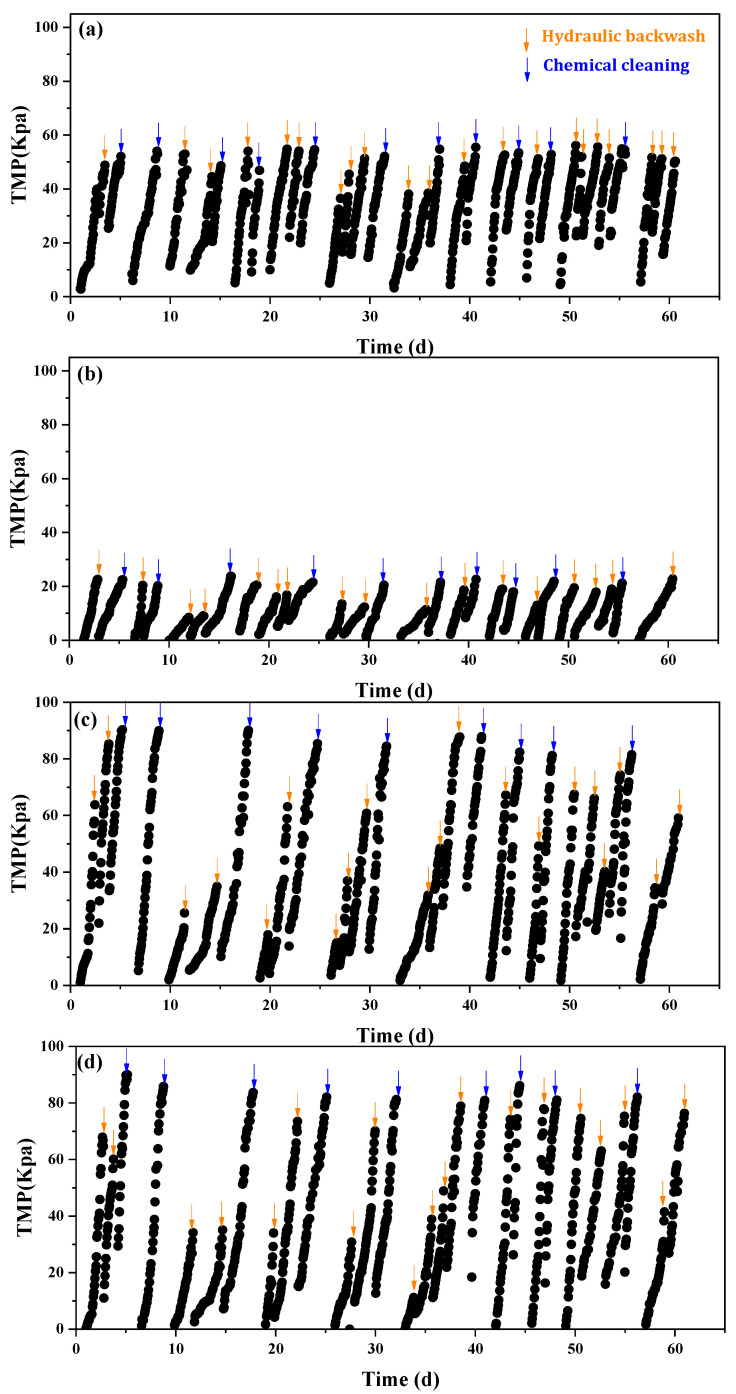
TMP changes of different membranes in the CMBR (**a**–**d**): C5, C7, C13, C20.

Figure 6 shows the trend of TMP development for the four corundum membranes with different pore sizes. During the long-term operation of the CMBR, hydraulic backwashing was carried out once a day; when the maximum TMP exceeded 80 kPa, chemical cleaning would be conducted for the four ceramic membranes. During the whole operation period, the TMP development rate of the four membranes was 24.73, 10.84, 33.83, and 34.22 kPa/d, respectively. Obviously, the C13 and C20 with larger pore sizes experienced the most severe membrane fouling during the MBR operation. The lowest membrane fouling was observed for the C7 membrane with a medium pore size of 0.63 μm. With the further decrease of membrane pore size to 0.50 μm, a higher TMP development rate was once again experienced by the C5 membrane. From this result, it was clear the C7 membrane possessed the optimum membrane pore size for alleviating membrane fouling in MBR. Moreover, the narrow pore size distribution of C7 relative to C13 and C20 might also make some contribution to its excellent antifouling performance.

**Figure 7:**
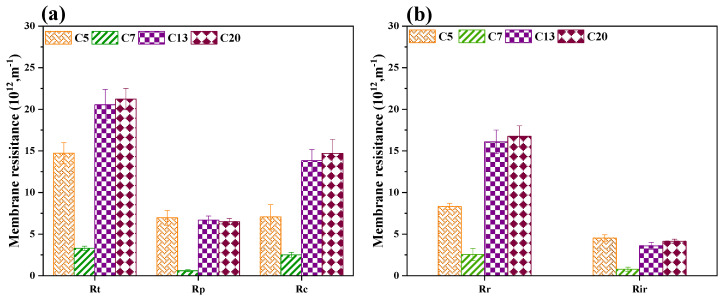
Distribution of membrane fouling resistances for different ceramic membranes in the CMBR: (**a**) pore blockage and cake layer resistances; (**b**) reversible and irreversible resistances.

Figure 7a shows the distribution of cake layer resistance (R_c_) and pore-blocking resistance (R_p_) for different membranes. It could be seen that for C5, the contribution of the cake layer and pore blockage to membrane fouling were essentially the same, while with the increase of membrane pore size, the proportion of cake layer resistance in total fouling resistance was significantly increased. The result manifested cake layer fouling played a dominant role in the membrane fouling of ceramic membranes with relatively larger pore sizes. The highest pore blockage fouling experienced by the C5 membrane might be attributed to its smallest pore size. Figure 7b displayed a detailed analysis of the reversible/irreversible fouling resistance (R_r_ and R_ir_). It could be seen that for all the ceramic membranes with different pore sizes, reversible fouling resistance was substantially higher than that of irreversible fouling, indicating the accumulation of foulants on the corundum membrane was highly reversible. In addition, both reversible and irreversible fouling of the C7 membrane was significantly lower than that of C5, C13, and C20, further demonstrating the C7 membrane possessed the optimum pore size that can effectively alleviate membrane fouling in MBR.

#### 3.3.2. Analysis of Cake Layer Foulants

**Figure 8:**
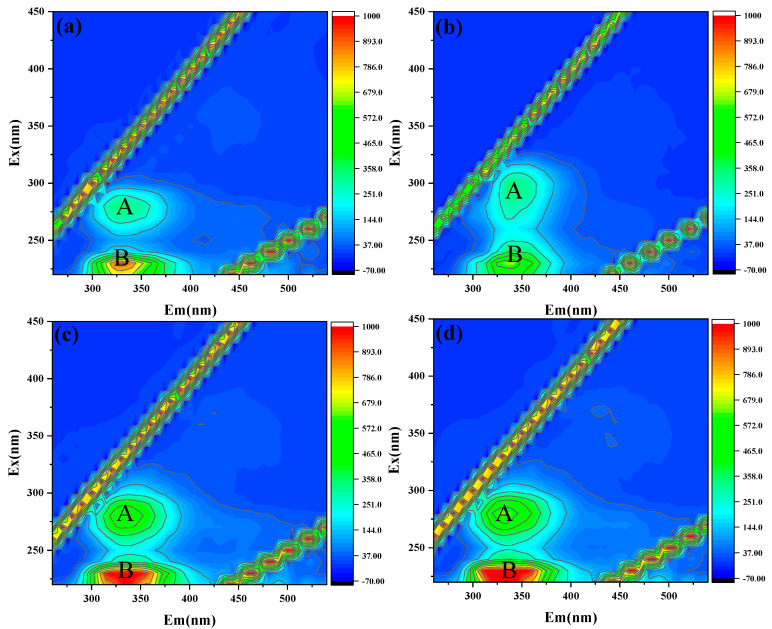
3D EEM of dissolved foulants extracted from (**a**–**d**) C5, C7, C13, C20. (peak A and B represent tryptophan-like substance and tyrosine-like substance, respectively).

Figure 8 displayed three-dimensional fluorescence spectra of the organic foulants extracted from the fouled membrane surface. Two distinct protein peaks were identified at 280/340 nm and 230/330 nm, which were associated with a tryptophan-like substance (peak A) and a tyrosine-like substance (peak B), respectively [32,33]. It could be seen that for both peak A and peak B, the fluorescence intensity followed the order of C20 > C13 > C5 > C7. This indicated that the concentration of organic foulants, especially the protein-like substances accumulated on the surface of C7, was the lowest. By contrast, the highest concentration of organic foulants was observed for the C20 with the largest pore size. This result was consistent with that in Section 3.3.1, i.e., the cake layer resistance of C5, C13, and C20 was much higher than that of C7.

**Figure 9:**
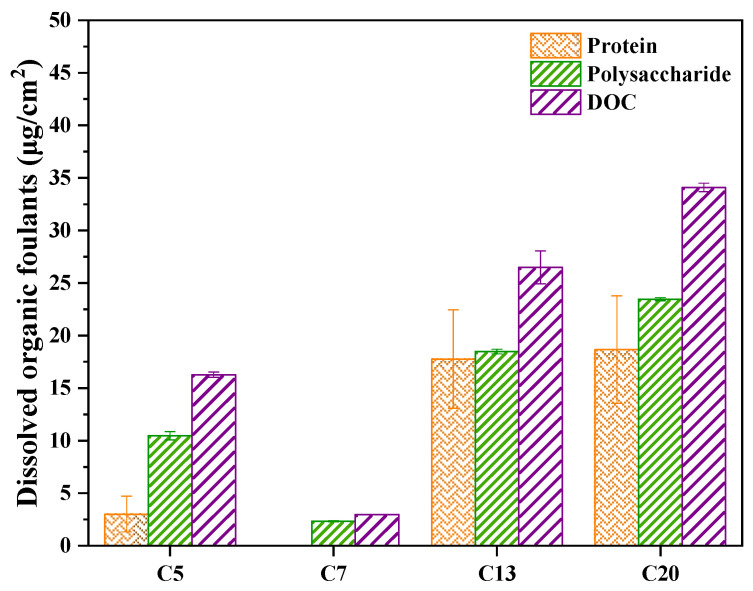
Protein, polysaccharide and DOC content on different membranes.

The protein, polysaccharide and TOC contents on the membrane surface were further quantified (Figure 9). Similar to the fluorescence peaks, the contents of protein, polysaccharide and DOC also followed the order of C20 > C13 > C5 > C7. Particularly, the protein content on the membrane surface of C7 was almost zero; the content of polysaccharides was also shown to be extremely low. Both protein and polysaccharide are the main constituent of EPS, which have been recognized as a critical factor for membrane fouling in MBR [34,35]. The result indicated that by optimizing membrane pore size, the accumulation of EPS on the ceramic membrane could be effectively inhibited, thus alleviating the membrane fouling during MBR operation.

#### 3.3.3. Fouled Membrane Morphology Analysis

**Figure 10:**
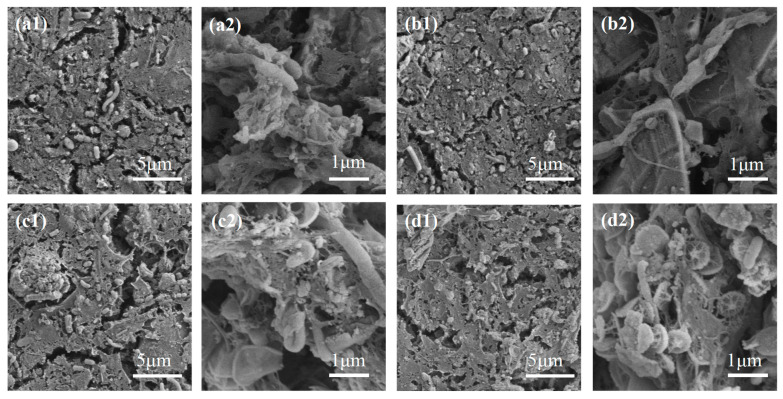
SEM images of the cake layer on the membranes with different pore sizes (**a1**,**a2**): C5 membrane; (**b1**,**b2**): C7 membrane; (**c1**,**c2**): C13 membrane; (**d1**,**d2**): C20 membrane).

Figure 10 shows the SEM images of the fouled ceramic membranes in the CMBR. From Figure 10(a1–d1), it can be seen that the surface of all membranes was covered with a dense cake layer, which was in coincidence with the resistance analysis, as shown in Figure 7. By careful inspection, it was observed the number of microorganisms gradually increased with the increase of membrane pore size. From the high-resolution images (Figure 10(a2–d2)), it could be seen that many flat circular bacteria and rod-shaped bacteria were attached to C5, C13, and C20. By contrast, much fewer microorganisms were present on C7, and only a few rod-shaped bacteria could be noticed. SEM images revealed the difference in the amount and species of microorganisms among the four membranes, which might be an important factor accounting for their difference in membrane fouling. Therefore, microbial communities in the cake layer were analyzed for the different membranes in the CMBR.

#### 3.3.4. Microbial Community Analysis

The result of Illumina HiSeq sequencing analysis is shown in Table 2. The coverage for the sludge samples taken from the fouled membranes and the ML in the CMBR was as high as 0.99, indicating that the measured sequences could truly and comprehensively reflect the microbial structure of the samples. The Chao1 index is usually used to estimate the variation in the number of OTUs, and the larger the number is, the richer the species source would be. The Shannon and Simpson indices are mainly used to compare microbial species diversity as well as homogeneity [36,37]. As can be seen, C7 exhibited relatively higher Shannon and Chao1 indices as well as a lower Simpson index in comparison with the other three ceramic membranes, implying that the species richness of C7 was slightly higher than other membranes. However, the total number of microorganisms on the membrane surface of C7 was smaller than the other three membranes, further verifying the cake layer on C7 possessed higher community diversity.

**Figure 11:**
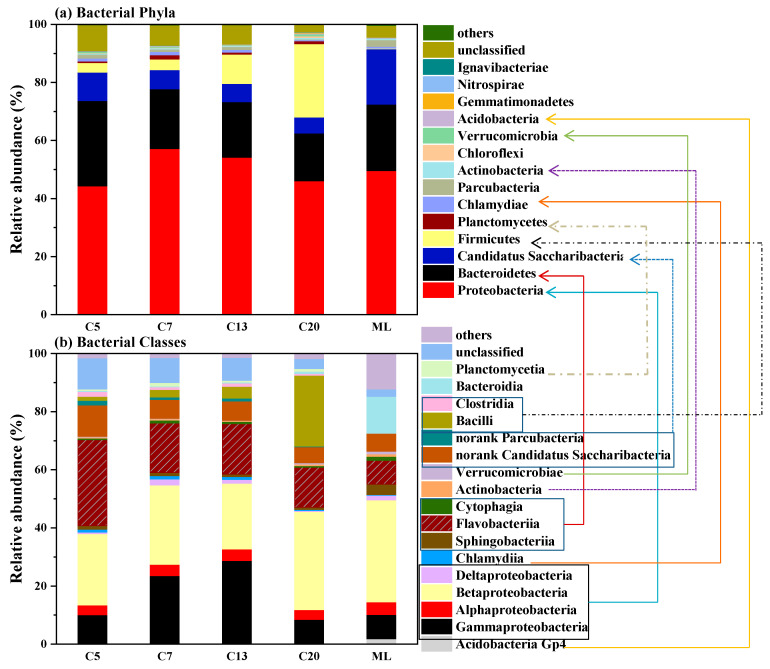
The relative abundance of the microbial community at the phylum level (**a**) and class level (**b**). (the arrows represent the bacterial classes belong to the corresponding bacterial phyla).

The effect of membrane pore size on the microbial community composition of the cake layer was further analyzed. Figure 11a showed *Proteobacteria*, *Bacteroidetes*, *Candidatus Saccharibacteria*, and *Firmicutes* were the four dominant phyla on the membrane surface. Among them, *Proteobacteria* was a common phylum of bacteria for nitrification and denitrification; while *Bacteroidetes* was able to produce proteins, an important component of EPS [38,39]. The relative abundance of *Bacteroidetes* was 32.3%, 20.6%, 20.0%, and 16.4% for C5, C7, C13, and C20, respectively. This might be one reason for the more severe membrane fouling experienced by C5 as compared with C7. Moreover, it had been reported that *Firmicutes* was a bacterial phylum often found in the cake layer and was extremely easy to adhere to the membrane surface [40]. The relative abundance of *Firmicutes* for the four ceramic membranes were 3.6%, 3.7%,10.6%, and 25.5%, respectively: i.e., the C7 possessed a much lower abundance of *Firmicutes* in its cake layer. Therefore, it was reasonable to consider the comprehensive effect of *Bacteroidetes* and *Firmicutes* phyla resulted in the severe membrane fouling of C5, C13, and C20.

Figure 11b shows the microbial community difference at the class level. Sixteen main bacterial classes were identified in the cake layer of the ceramic membranes. Among them, *Alphaproteobacteria*, *Gammaproteobacteria, Betaproteobacteria,* and *Deltaproteobacteria* were subordinated to *Proteobacteria*; while *Sphingobacteriia*, *Cytophagia,* and *Flavobacteriia* belonged to *Bacteroidetes*. C5 had the highest relative abundance in *Flavobacteriia* (29% vs. 17.1%, 17.3%, and 13.7% for C7, C13, and C20, respectively), which was reported to be involved in EPS secretion and was closely associated with membrane fouling [41]. This observation was in coincidence with that made at the phylum level. In addition, extensive research has shown that in the MBR system, *Bacilli* bacteria played a vital role in the cake layer fouling [42], which could produce proteins in the EPS and thus drove the formation and development of biofilm with strong adhesion properties [43]. It was interesting to observe that the relative abundance of *Bacilli* was significantly increased with the increase of membrane pore size. Its relative abundance on C13 (4.1%) and C20 (24.4%) was 1.6 and 9.4 times higher than that on C7 (2.6%). This could be an important reason for the more severe membrane fouling witnessed by C13 and C20. The result demonstrated once again that membrane pore size had an important influence on the microbial community structure in the cake layer, and by optimizing the membrane pore size, the membrane biofouling could be efficiently alleviated.

## 4. Conclusions

In this work, hollow flat-sheet ceramic membranes with different pore sizes were prepared using corundum with different grain sizes. The obtained membranes were systematically characterized, and the membrane fouling behavior in MBR was studied. The following conclusions could be drawn.

(1) With the increase of corundum grain size, the mean membrane pore size, porosity and pure water flux were shown to be increased. Correspondingly, the uniformity of pore size distribution was decreased, with the appearance of macropore defects.
(2) C7, with a medium pore size (0.63 μm), exhibited the lowest TMP development rate. It was interesting that with the increase of membrane pore size, cake layer fouling became more dominant in the total membrane fouling of the ceramic membrane.
(3) The content of protein, polysaccharide and DOC accumulated on the membrane surface of C7 was substantially lower than that on the other three membranes, further demonstrating the antifouling ability of the C7 membrane.
(4) Compared with the other three membranes, C7 had a lower relative abundance of Bacteroidetes and Firmicutes at the phylum level and a lower relative abundance of Flavobacteria and Bacilli at the class level, which could slow down the formation and development of biofouling on the membrane surface.

## Figures and Tables

**Table 1:** Characteristics of the ceramic membranes.

Membrane Samples	Mean Pore Size (μm)	Porosity (%)	Pure Water Flux (L/(m^2^∙h∙bar))	Crystallite Size (nm)
C5	0.50	41.43	2111.67	722
C7	0.63	45.44	6016.20	749
C13	0.80	46.31	8309.16	758
C20	1.02	47.60	15,193.08	951

**Table 2:** The diversity estimators of microbes for different samples.

Sample	Number	OTUs	Shannon	Chao 1	Simpson	Coverage
C5	36,026	768.0	3.78	946.93	0.07	0.99
C7	33,600	787.0	3.99	944.50	0.06	0.99
C13	35,726	733.0	3.78	938.15	0.06	0.99
C20	39,868	749.0	2.97	924.47	0.17	0.99
ML	29,814	380.0	0.78	380.0	0.03	0.99

## Data Availability

The data presented in this study are available in the article.
